# Size and Carbon Content of Sub-seafloor Microbial Cells at Landsort Deep, Baltic Sea

**DOI:** 10.3389/fmicb.2016.01375

**Published:** 2016-08-31

**Authors:** Stefan Braun, Yuki Morono, Sten Littmann, Marcel Kuypers, Hüsnü Aslan, Mingdong Dong, Bo B. Jørgensen, Bente Aa. Lomstein

**Affiliations:** ^1^Center for Geomicrobiology, Department of Bioscience, Aarhus UniversityAarhus, Denmark; ^2^Geomicrobiology Group, Kochi Institute for Core Sample Research, Japan Agency for Marine-Earth Science and TechnologyKochi, Japan; ^3^Biogeochemistry Group, Max Planck Institute for Marine MicrobiologyBremen, Germany; ^4^Interdisciplinary Nanoscience Center, Aarhus UniversityAarhus, Denmark; ^5^Section for Microbiology, Department of Bioscience, Aarhus UniversityAarhus, Denmark

**Keywords:** cell volume, carbon content, carbon density, cell extraction, FACS, IODP, Expedition 347, deep biosphere

## Abstract

The discovery of a microbial ecosystem in ocean sediments has evoked interest in life under extreme energy limitation and its role in global element cycling. However, fundamental parameters such as the size and the amount of biomass of sub-seafloor microbial cells are poorly constrained. Here we determined the volume and the carbon content of microbial cells from a marine sediment drill core retrieved by the Integrated Ocean Drilling Program (IODP), Expedition 347, at Landsort Deep, Baltic Sea. To determine their shape and volume, cells were separated from the sediment matrix by multi-layer density centrifugation and visualized via epifluorescence microscopy (FM) and scanning electron microscopy (SEM). Total cell-carbon was calculated from amino acid-carbon, which was analyzed by high-performance liquid chromatography (HPLC) after cells had been purified by fluorescence-activated cell sorting (FACS). The majority of microbial cells in the sediment have coccoid or slightly elongated morphology. From the sediment surface to the deepest investigated sample (~60 m below the seafloor), the cell volume of both coccoid and elongated cells decreased by an order of magnitude from ~0.05 to 0.005 μm^3^. The cell-specific carbon content was 19–31 fg C cell^−1^, which is at the lower end of previous estimates that were used for global estimates of microbial biomass. The cell-specific carbon density increased with sediment depth from about 200 to 1000 fg C μm^−3^, suggesting that cells decrease their water content and grow small cell sizes as adaptation to the long-term subsistence at very low energy availability in the deep biosphere. We present for the first time depth-related data on the cell volume and carbon content of sedimentary microbial cells buried down to 60 m below the seafloor. Our data enable estimates of volume- and biomass-specific cellular rates of energy metabolism in the deep biosphere and will improve global estimates of microbial biomass.

## Introduction

The correct determination of bacterial cell size and biomass is critical for understanding many aspects of microbial ecology. For pure cultures and natural ecosystems on Earth's surface, both parameters have been determined for different levels of nutrient availability (e.g., Bratbak, 1985; Kogure and Koike, 1987; Simon and Azam, 1989; Fagerbakke et al., 1996; Troussellier et al., 1997; Fukuda et al., 1998; Romanova and Sazhin, 2010; Lever et al., 2015). However, little is known about size and biomass of cells in Earth's energy-limited subsurface, such as the extensive marine sediment.

The marine deep biosphere is cut off from surface energy supplies over geologic time scales. Microbes have to efficiently use the limited chemical potential energy in the sediment for producing ATP and synthesizing new biomass. Our knowledge of how microbial communities adapt to long-term subsistence at very low energy availability in the deep seabed remains fragmentary (Lever et al., 2015). Measuring cell size and biomass may help understand whether subsurface microorganisms physically adapt to the harsh conditions faced during burial. In addition, both parameters are important for estimating the significance of deep microbial communities in the global carbon cycle.

Bacterial cell size and biomass in the deep biosphere are poorly constrained possibly because the complex sediment matrix interferes with the analysis of whole cells and sub-cellular compounds. For accurate measurements of cell volume and cellular organic carbon, cells must first be separated from mineral grains and detrital organic particles, which is extremely difficult and time-consuming (Kallmeyer et al., 2008; Morono et al., 2013). Consequently, there is hardly any data on the size and cellular carbon content of cells in the deep biosphere.

Estimates of the total amount of microbial biomass (i.e., cellular organic carbon) in the seabed range from 4 to 300 Pg C (Parkes et al., 1994; Whitman et al., 1998; Lipp et al., 2008; Kallmeyer et al., 2012). However, these estimates were not based on direct measurements of the carbon content of sub-seafloor microbial cells. Instead, they were based on mean cellular carbon contents that were deduced either from mean literature values (Parkes et al., 1994), cell dry weight (Whitman et al., 1998), sedimentary lipid biomarker concentrations (Lipp et al., 2008), or cell volumes (Kallmeyer et al., 2012). While the cell volumes and the lipid concentrations were quantified from actual sub-seafloor samples, the data for the dry weight consisted of one single data point obtained from a terrestrial aquifer (Balkwill et al., 1988). These parameters were then converted into cellular carbon contents using relationships determined for pure cultures or planktonic bacteria from water samples (e.g., Norland et al., 1987; Balkwill et al., 1988; Simon and Azam, 1989).

In this study, we quantified the cell volume and amino acid-carbon content of cells from a sediment drill core retrieved during IODP Expedition 347 in October 2013 at Landsort Deep, Baltic Sea (Expedition 347 Scientists, 2014). The drilling site was situated in the central part of the Landsort Deep, which is the deepest sub-basin (437 m water depth) in the Baltic Sea Basin. It contains a thick and continuous record of the last ~14,000 years, including the transition from Holocene, organic-rich clay to glacial, low-organic clay (Andrén et al., 2015). It is characterized by high sedimentation rates (100–500 cm kyr^−1^), high concentrations of total organic carbon (TOC), and extremely high microbial abundance (up to 10^10^ cells cm^−3^) in the organic-rich Holocene deposits (Andrén et al., 2015).

Cell sizes were determined by epifluorescence microscopy (FM) and scanning electron microscopy (SEM) after cells had been separated from the sediment matrix by multi-layer density centrifugation (Morono et al., 2013). Because of the relatively weak relationship between cell volume and carbon content (Romanova and Sazhin, 2010), the latter was independently estimated in seven samples that were purified by fluorescence-activated cell sorting (Morono et al., 2013; Braun et al., 2016). To correct the FM-based cell sizes for the fluorescence halo-effect (when aureoles appear around the cell), we used correction factors obtained from volume measurements of cultured *Escherichia coli* and *Micrococcus luteus* cells by FM and atomic force microscopy (AFM). The cultured cells were also used to test whether the filtration of cells onto membrane filters affects the cell volume. Furthermore, literature values were used to correct for shrinkage due to cell fixation and critical point drying. Finally, the cell-specific carbon content was determined from direct measurements of cellular amino acids and by assuming that these contain ~55% of total cell carbon (Ingraham et al., 1983).

Given the large extent of marine sediment on Earth, assessing the size and carbon content of sub-seafloor microbial cells will improve global estimates of microbial biomass and carbon turnover.

## Materials and methods

### Samples

A 120-m long sediment core was taken by piston core drilling during IODP Leg 347 at Landsort Deep (58°37.34 N, 18°15.25 E; Site 63, Hole E) at 437 m water depth (Andrén et al., 2015). Perfluorocarbon (PFC) tracer was used while drilling to evaluate potential contamination of microbiology samples with cells from the drilling fluid. The average contamination level corresponded to the potential introduction of 10–100 cells cm^−3^ of sediment (Andrén et al., 2015). In comparison to the *in-situ* cell abundance of 10^8^–10^10^ cells cm^−3^, this was still less than a millionth of the indigenous community.

Sediment for cell extraction (~5 cm^3^) was sub-sampled from whole-round core sections with sterile cut-off syringes and stored at −80°C until further processing. For method development, we also used three surface sediment samples taken with a Rumohr corer during Expedition SA13 on the continental shelf in the Labrador Sea (64°26.74 N, 52°47.65 W) at a water depth of 498 m in August 2013. Those three samples were placed in sealed airtight plastic bags along with an oxygen consuming pack (AnaeroGen, Oxoid, Roskilde Denmark) and stored anoxically at 4°C to keep cells intact. Cultures of *E. coli* (DSM 498) and *M. luteus* (DSM 20030) were grown in nutrient broth medium at 37°C and harvested in late exponential phase. Cultured cells were then fixed in paraformaldehyde (PFA, 2% final concentration) for 6 h at 4°C, then washed 3 × in phosphate-buffered saline (PBS), resuspended in PBS:ethanol 1:1, and stored at −20°C.

### Cell separation

All materials and reagents were filter-sterilized (0.2 μm pore size) and/or autoclaved before use. To separate intact microbial cells from the sediment matrix, we performed density gradient centrifugation on slurried sediment. Sediment (0.5 cm^3^) was fixed in PFA (2% final concentration) for 6 h at 4°C, then washed 3 × in PBS and resuspended in PBS:ethanol 1:1 in 15-mL Falcon tubes and stored at −20°C. Cell extraction was then performed based on the protocol of Morono et al. (2013). Fixed sediment slurries were centrifuged at 5000 × g for 5 min, after which the supernatant was discarded. The pelleted sediment was resuspended in 1.5 mL Milli-Q water that included 0.2 mL methanol and 0.2 mL detergent mix (consisting of 100 mM EDTA, 100 mM sodium pyrophosphate decahydrate, and 1% v:v Tween 80). Samples were then shaken for 60 min at 750 rpm. After shaking, the samples were sonicated for 3 × 15 s using an ultrasonic probe (14 W). To establish a density gradient, three layers of Nycodenz (30%, 50%, 80% w:v; 2 mL each layer; Nycodenz from AXIS-SHIELD PoC AS, Oslo, Norway) were injected beneath the sediment slurry using a syringe with a long needle. A 2-mL layer of sodium polytungstate solution (Sometu, Berlin, Germany) with a density of 2.23 g mL^−1^ was added beneath the Nycodenz layers. Samples were then centrifuged at 5000 × g for 2 h at 4°C. After centrifugation, the supernatant above the sodium polytungstate solution was removed with a pipette and kept as “cell extract” in sterile Falcon tubes. Extraction efficiencies (i.e., cell recoveries) were calculated from the number of extracted cells per volume of sediment and the total number of cells per volume of sediment. Enumeration of cells in whole sediment is described in see Section Cell Enumeration. Extraction efficiencies were between ~5 and 50%.

It has previously been shown that density-based extraction of cells from sediment is representative of the *in-situ* community at a taxonomic level (Frischer et al., 2000; Braun et al., 2016). To test whether extraction of cells was also representative in terms of cell volume and cell morphology, we compared extracted cells to those in whole sediment in three samples from Landsort Deep and one sample from the Labrador Sea (Supplementary Figure 1). Cell morphology was similar between extracted and non-extracted cells, but the relative amount of coccoid cells was slightly higher in the sediments than in the cell extracts (Supplementary Figure 1A). However, the cell volumes of extracted and non-extracted cells followed a 1:1 line (Supplementary Figure 1B), indicating that extraction of cells from sediment was representative in terms of size.

For measurements of cellular amino acid contents, cell separation was performed as described by Morono et al. (2013). Cell extracts from density centrifugation were then subjected to extensive cell purification using FACS to remove detrital particles as described in Braun et al. (2016).

### Cell enumeration

Epifluorescence microscopy cell counts were performed on bulk sediment and on cell extracts after cell separation from the sediment matrix. For direct counts (bulk), we usually suspended 0.5 cm^3^ of sediment in 2–15 o NaCl solution (approximating the salinity of the sample) with 2% PFA, followed by filtration (0.2-μm pore size polycarbonate membrane filter, Millipore GmbH, Eschborn, Germany) of a small aliquot of the slurry (tens of μL depending on the cell density) and staining with 4′,6-diamidin-2-phenylindol (DAPI). Aliquots (10–250 μL) of cell extracts that had been separated from the sediment were diluted in ~5 mL Milli-Q water and then directly collected on a polycarbonate membrane filter (0.2-μm pore size) and stained with DAPI. Cells were manually enumerated under an epifluorescence microscope. Generally, either 30 fields of view or at least 200 cells were counted at 1000 × magnification.

### Cell size determination with FM

Cell size measurements were performed on the same membrane filters used for cell enumeration. Cell sizes were determined on images acquired with the software AxioVision (Carl Zeiss MicroImaging GmbH, Göttingen, Germany) under 1000 × magnification. Since the actual cell boundary could not accurately be distinguished from the surrounding fluorescence-halo, cell lengths, and widths were measured including the fluorescence-halo.

### Cell size determination with SEM

Aliquots (10–250 μL) of cell extracts were diluted in ~5 mL Milli-Q water and filtered onto gold-sputtered polycarbonate membrane filters (0.2-μm pore size, 25 mm diameter, Millipore GmbH, Eschborn, Germany). To keep the cells hydrated, ~1 mL of the liquid was left on the membrane before it was quickly transferred into 30% ethanol (in Milli-Q water). The membrane was then subjected to an ethanol-series (30, 50, 70, 80, 99% ethanol) with 10 min residence time per concentration. To dry the cells on the filter while retaining their shape, the filter was transferred into a critical point dryer (Leica EM CPD 300, Wetzlar, Germany, slow gas in/out, 14 cycles, heating 40°C). The ethanol was exchanged with liquid CO_2_ followed by evaporation of the CO_2_ at the critical point. Samples were then analyzed by SEM (Quanta FEG 250, FEI, Eindhoven, The Netherlands) under high vacuum conditions using an Everhart-Thornley detector (ETD) for secondary electron (SE) imaging and an acceleration voltage of 2 kV for the electron beam. For the imaging process, 128 images were captured each with a dwell time of 100 ns, drift corrected and integrated. Cell sizes were measured on images using the software FEI xT Microscope Control (FEI GmbH, Frankfurt, Germany).

To confirm that cells visualized with SEM retained their 3-dimensional shape after dehydration and critical point drying, we took stereoscopic images as well as images before and after tilting the sample table to up to 40° (Supplementary Figure 2). Even though we cannot exclude that cells have shrunken uniformly due to dehydration and critical point drying, they did not show any signs of flattening, which would have biased the lengths and widths measurements (and eventually the volume, since we assumed width = height).

### Cell size determination with AFM

For details on the sample preparation, see Section Corrections for Sample Treatments. DimesionIcon (Bruker, Santa Barbara, USA) AFM was used in PeakForce Tapping™ Mode for structural characterization of the samples. Operation was conducted using triangular silicon nitride cantilever (ScanAsyst Air) with 70 kHz nominal resonant frequency, 0.4 N/m nominal spring constant, and 2 nm nominal tip radius. Cantilevers were calibrated before use. Operation parameters were set in order to achieve highest possible resolution without damaging the sample or the tip. AFM images of 512 points per line with various scan sizes were recorded at a scan rate of 0.5–1 Hz. Multiple images of the same sample from different locations were collected for the best statistical overview. Collected images were processed and analyzed using the open source software Gwyddion (www.gwyddion.net).

### Calculations of cell volumes and cell-specific carbon densities

FM-based cell volumes were calculated for the bacterial shapes coccus, rod, and filament. SEM-based cell volumes also included volumes for prolate spheroids. AFM-based cell volumes, which take into account the individual height of the cells, were calculated based on ellipsoids. The mathematical formulations can be found in the Supplementary Information.

The cell-specific carbon density, *C*_*d*_, was calculated by dividing the cell-specific carbon content of a sample by the mean FM-based cell volume. The mean cell volume was determined from the fractions (*f*) and mean volumes (*V*) of coccoid, rod-shaped, and filamentous cells per sample according to the following equation:

$$\begin{array}{l} C_{d}=\frac{\text{Cell-specific carbon content}}{V_{coccus}\times f_{coccus}+V_{rod}\times f_{rod}+V_{filament}\times f_{filament}} \end{array}$$

### Corrections for sample treatments

Cell sizes were corrected for the following sample treatments or size estimation bias: filtration, fluorescence-halo, fixation, and critical point drying (Table 1). While correction factors for fixation and critical point drying were obtained from the literature, those for filtration and for the fluorescence halo-effect were experimentally determined on cultured *E. coli* and *M. luteus* cells.

**Table 1:** **Correction factors applied to cell volumes obtained from FM and SEM**.

**Sample**	**Fixation**	**Filtration**	**Halo-effect**	**Dehydration and CPD**
**FM-BASED**				
Coccoid	1.29^*^	1	0.475	n.a.
Elongated	1.29^*^	1.13	0.475	n.a.
Filamentous	1.29^*^	1.13	0.475	n.a.
**SEM-BASED**				
Coccoid	1.29^*^	1	n.a.	2.38^**^
Elongated	1.29^*^	1.13	n.a.	2.38^**^
Filamentous	1.29^*^	1.13	n.a.	2.38^**^

* *Average of values given by Bowden (1977) and Fagerbakke et al. (1996)*.

** *Average value taken from Bratbak (1985) and references therein*.

*n.a., not applicable*.

To obtain a correction factor to account for volume shrinkage from the filtration of cells onto filter membranes, we used fixed (2% PFA for 6 h at 4°C) *E. coli* and *M. luteus* cells. Immediately before the experiment, cells were washed 3 × with Milli-Q water. Aliquots of the cell suspensions were filtered onto gold-sputtered polycarbonate membrane filters (0.2-μm pore size), whereas few drops (~30 μL) of the cell suspensions were pipetted onto Si-wafers coated with polylysine to immobilize cells on the wafer. When applying drops of cell suspensions onto Si-wafers, the wafers were left in Petri-dishes for about an hour, which allowed cells to settle onto the wafer. The wafer was then gently rinsed with Milli-Q water and transferred into 30% ethanol solution. During the procedure, great care was taken to ensure that cells on the membrane filters and Si-wafers never dried out and were always immersed in a small amount of liquid. Filters and Si-wafers were then subjected to an ethanol-series and critical point drying as described above. Cell sizes and volumes were calculated from SEM images of ~100 individual cells for each sample. There was no significant difference between the mean cell volumes of filtered and non-filtered *M. luteus* cells (*P* = 0.1307), but significant volume shrinkage of 11.2% for *E. coli* cells (*P* = 0.0408). The correction factor for the filtration step was then obtained by dividing the mean cell volumes by each other. For example, the mean cell volume of filtered *E. coli* cells was 0.68 μm^3^, while that of non-filtered *E. coli* cells was 0.76 μm^3^. The cell volumes of filtered elongated cells (rods and filaments) from the sediment samples were therefore corrected by the factor 0.76/0.68 = 1.13 (Table 1).

To obtain a correction factor for the fluorescence halo-effect (when aureoles appear around the cell; cf. Schumann and Rentsch, 1998), cell suspensions of the cultured *E. coli* and *M. luteus* cells were filtered onto polycarbonate membrane filters (0.2-μm pore size). The filters were then cut into two halves, one of which was subjected to DAPI-staining and FM imaging, while the other half was mounted onto mica for AFM imaging. AFM imaging required additional blank membrane filters for background correction. Cell sizes and volumes were calculated from FM and AFM images of ~100 individual cells for each sample. Including the fluorescence-halo into the cell size estimate of *E. coli* cells increased the calculated volume by a factor of 2.1 (cell volumes were therefore corrected by the factor 1/2.1 = 0.48, Table 1). Unfortunately, aggregation of *M. luteus* cells made size determination with FM impossible. Coccoid cells were therefore corrected with the factor obtained for *E. coli* cells.

**Figure 1:**
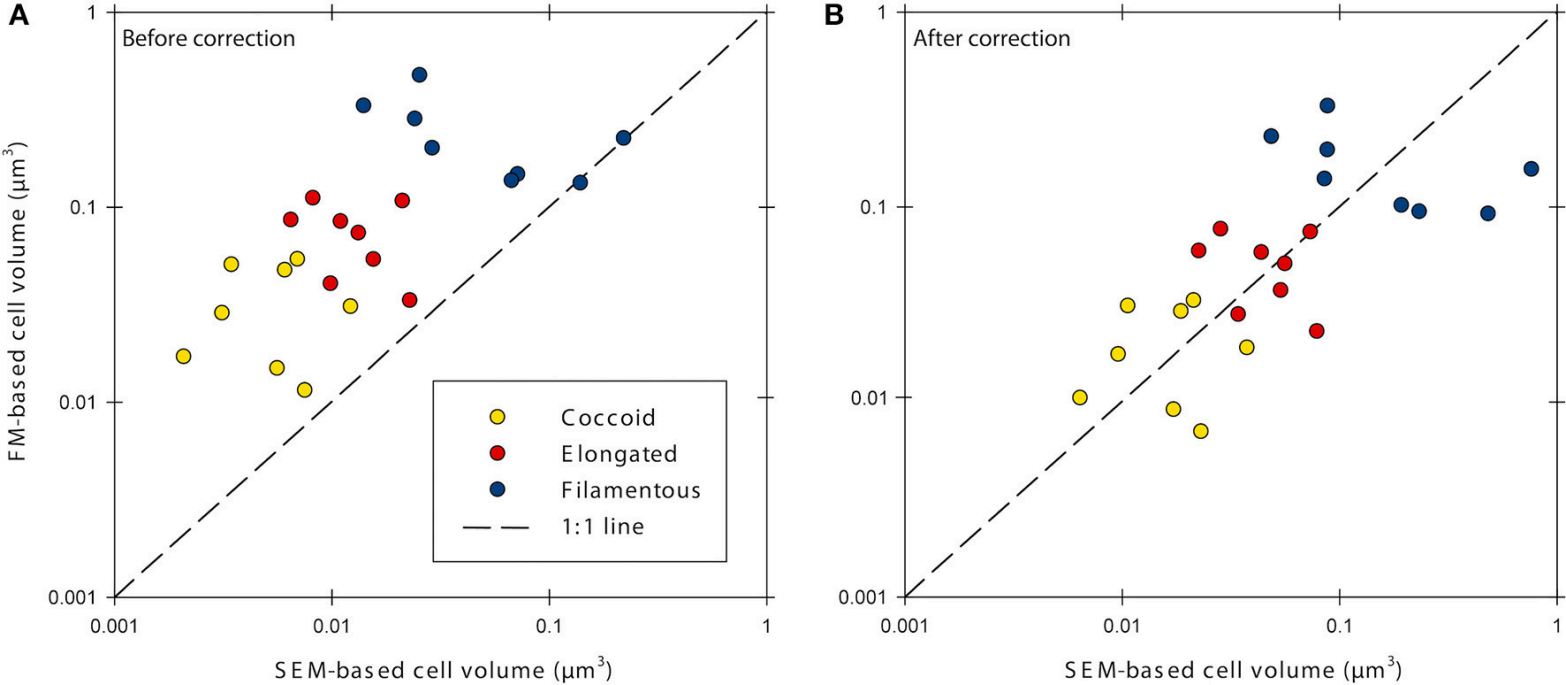
**Cell volumes determined with epifluorescence microscopy (FM) and scanning electron microscopy (SEM) before (A) and after (B) correction for errors caused by sample processing**. Data was corrected for cell fixation, filtration, dehydration and critical point drying, and the fluorescence-halo effect.

Cell volumes were corrected for cell fixation using the average (22.5% volume shrinkage) of values given by Bowden (1977) and Fagerbakke et al. (1996). Finally, we corrected for volume shrinkage due to dehydration with ethanol followed by critical point drying by using an average value for shrinkage of 58% taken from Bratbak (1985) and references therein. We then corrected the cell volumes from the investigated sediment samples with the corresponding correction factors (Table 1, Figure 1). Elongated and filamentous cells were corrected with factors obtained for *E. coli* cells. Coccoid cells in the samples were corrected with factors obtained for *M. luteus* cells with the exception of the halo-effect, which was corrected with the factor obtained for *E. coli* cells because aggregation of *M. luteus* cells made size determination with FM impossible. After correction, cell volumes obtained with FM were similar to those obtained with SEM (Figure 1B, Supplementary Figure 3).

### HPLC analysis of cellular amino acids

HPLC analyses of total hydrolyzable amino acids (THAA) were performed in a special clean lab at the Department of Bioscience, Aarhus University, Denmark. The temperature in the clean lab is constantly held at 21°C and HEPA air filters are integrated into the ventilation system. Ultra-clean Milli-Q-water (Milli-Q Integral 3, Millipore) as well as HPLC-grade reagents were used for analysis. All materials were acid-washed (1 N HCl) prior to use, and all pipettes/tips were calibrated by weighing 0.005–1 mL aliquots of Milli-Q water with an accuracy of ±0.0003 g prior to pipetting of analytes. The concentrations of THAA in FACS-purified cell extracts on ADVANTEC membrane filters and in blank ADVANTEC membrane filters were analyzed by reverse-phase HPLC (Waters Corporation, Eschborn, Germany) of fluorescent o-phthaldialdehyde (OPA)-derivatized products according to the method of Lindroth and Mopper (1979) and with the modifications described in Langerhuus et al. (2012). Briefly, membrane filters were hydrolyzed with 5 mL 6 N HCl at 105°C for 24 h under N_2_. Subsamples (4.5 mL) of hydrolyzate were dried under vacuum at 45°C, then re-suspended in Milli-Q water, and dried again. Dried samples were then dissolved in 2 mL Milli-Q water and filtered (0.2-μm pore size filter; Sartorius). The columns used were a Waters Nova-Pak® guard column (4 μm; 3.9 × 20 mm) followed by a Waters Nova-Pak® C-18 (4 μm; 3.9 × 150 mm) column. Blanks were always prepared along with samples. Blanks that were analyzed along with sediment samples showed negligible target molecule concentrations. Blanks that were prepared and analyzed along with cell extract samples had slightly higher target molecule concentrations, which were subtracted from those in cell extract samples.

## Results

### Cell shapes and cell volumes

**Figure 2:**
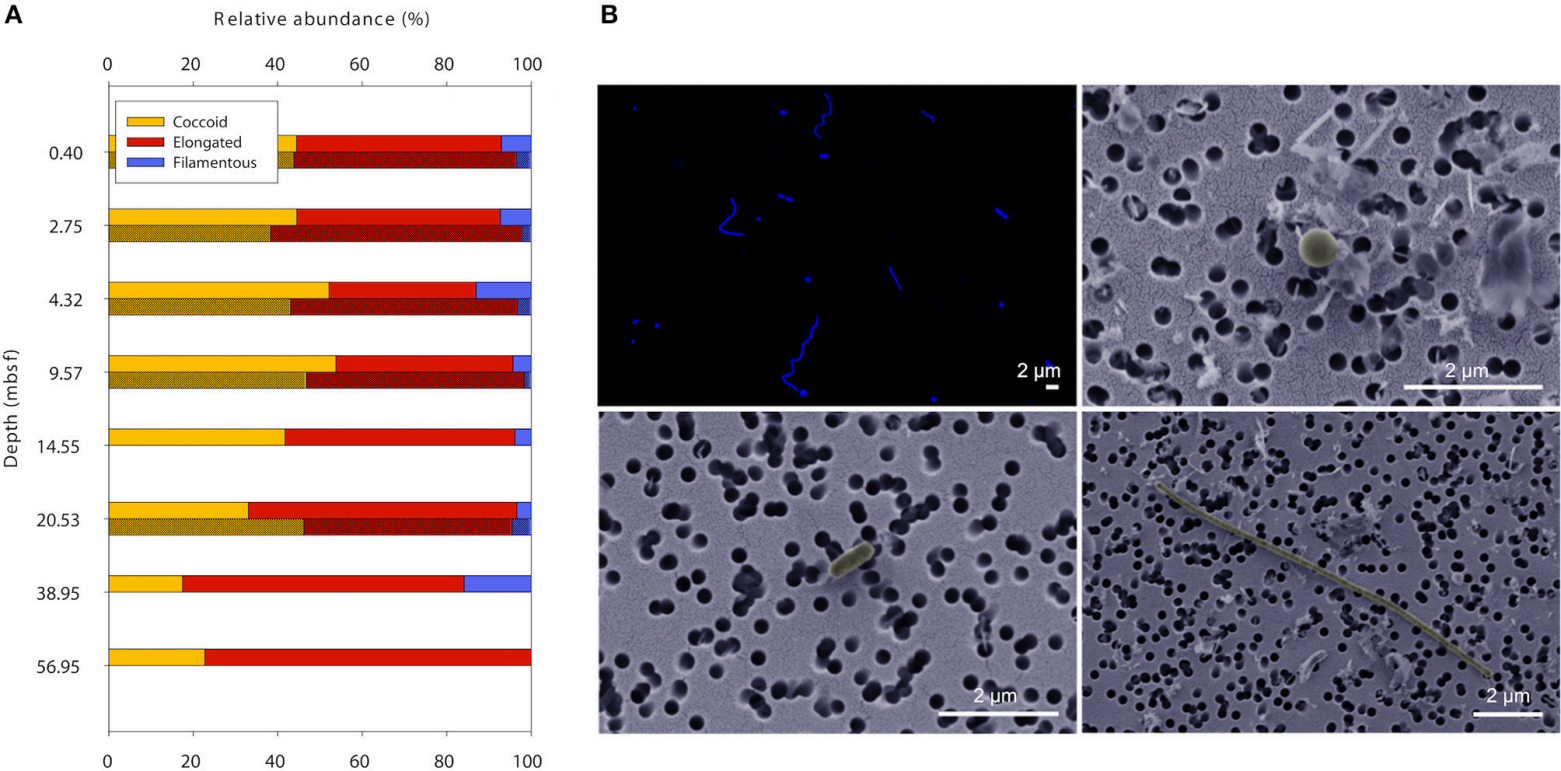
**Cell shapes throughout 60 meters of sediment at Landsort Deep. (A)** The majority of microbial cells in the sediment have coccoid or slightly elongated morphology, whereas filamentous cells are rather scarce. Data obtained with FM (lighter shade) and SEM (darker shade). **(B)** Typical images of DAPI-stained cells seen with FM (upper left), and coccoid (upper right), elongated (lower left), and filamentous cells (lower right) seen with SEM.

For simplification and better comparability between methods, rod-shaped and prolate spheroid cells were grouped as elongated cells. Both methods FM and SEM showed that the majority of cells in the sediment samples were of coccoid or slightly elongated morphology with a length-to-width ratio between 1 and 3 (Figure 2). Long filamentous cells (length:width ratio >10) were only a minor part of the microbial communities (<10%).

**Figure 3:**
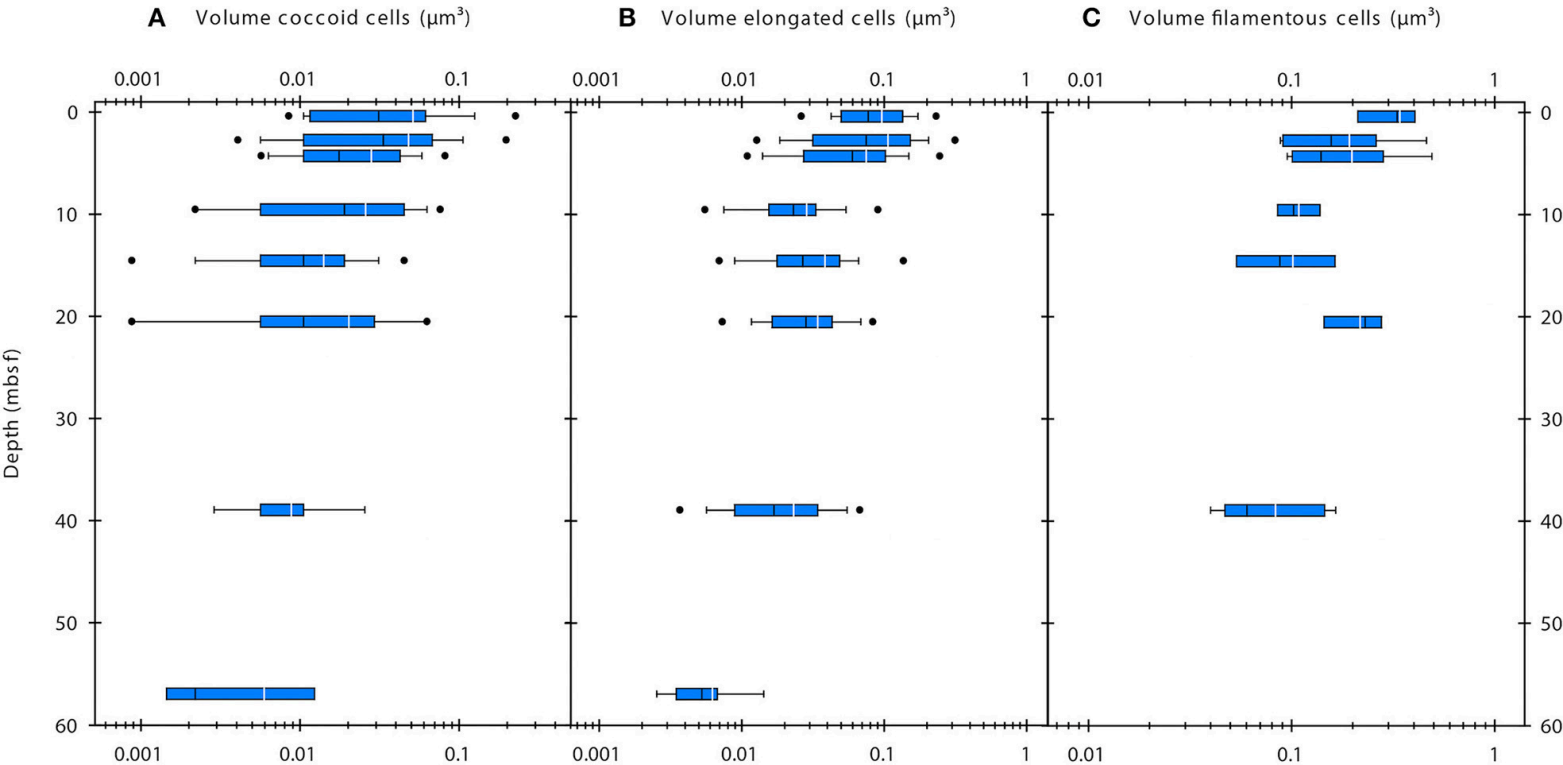
**Volumes of coccoid (A), elongated (B), and filamentous cells (C) determined by epifluorescence microscopy**. White line indicates mean value, black line indicates median value. The blue boxes indicate the 25th/75th percentile. Whiskers show 10th/90th percentile, dots show 5th/95th percentile.

SEM imaging of cells was only successful for a subset of the samples. In sediment samples with cell abundances of < 5 × 10^8^ cells cm^−3^, cell densities on the filter membranes were extremely low and the lack of a fluorescence signal made it extremely difficult to find cells at the high resolution used during SEM imaging. Therefore, we used the corrected FM-based cell volumes for further data presentation and interpretation. The average corrected cell volumes of coccoid and elongated cells significantly decreased with depth (Single factor ANOVA; coccoid cells, *F* = 23.32, *df* = 287, *P* = 2.232 × 10^−6^; elongated cells, *F* = 73.35, *df* = 382, *P* = 2.699 × 10^−16^) from ~0.05 to 0.006 μm^3^ and from ~0.1 to 0.006 μm^3^, respectively (Figures 3A,B). The cell volumes of filamentous cells significantly decreased with depth (Single factor ANOVA; *F* = 9.815, *df* = 46, *P* = 0.003009) from ~0.3 to 0.08 μm^3^ (Figure 3C). Within samples, the cell volumes of coccoid and elongated cells typically varied within one order of magnitude, whereas those of filamentous cells varied about half an order of magnitude.

Cell volumes were also determined for three surface sediment samples from the Labrador Sea, where cell abundance was high (>10^9^ cells gdw^−1^) and samples were stored anoxically at 4°C instead of −80°C to ensure cell integrity. The data for Labrador Sea samples are included in Figure 1 and show a similar pattern as samples from Landsort Deep (see also Supplementary Figure 3B).

### Cell-specific carbon content and carbon density

**Figure 4:**
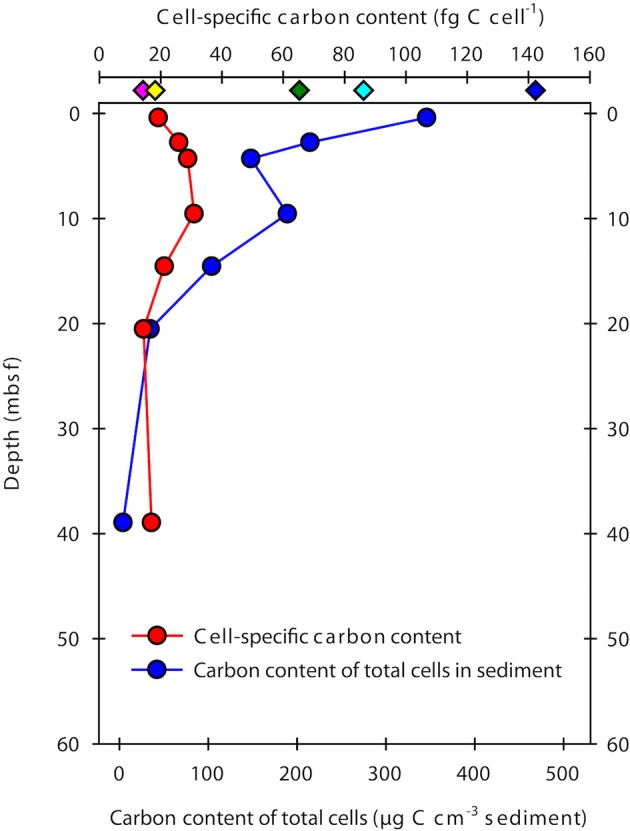
**Cell-specific carbon content and total microbial cell carbon in samples from Landsort Deep**. The cell-specific carbon content (red symbols) was determined from direct measurements of cellular amino acids, assuming these comprise ~55% of total cell carbon (Ingraham et al., 1983). Diamond symbols at the top indicate previously estimated cellular carbon contents for sub-seafloor cells (green, Parkes et al., 1994; cyan, Whitman et al., 1998; yellow, Lipp et al., 2008; pink, Kallmeyer et al., 2012) and for FACS-purified *E. coli* cells (blue, Braun et al., 2016). The carbon content of total microbial cells in the sediment (blue circle symbols) was obtained by multiplying the mean cell-specific carbon content with the number of DNA stainable microbial cells (Andrén et al., 2015).

The cell-specific carbon content was determined for seven samples from Landsort Deep from direct measurements of cellular amino acids (Tables 2, 3). By assuming that amino acid-carbon comprises ~55% of total cell carbon (Ingraham et al., 1983), the calculated mean cell-specific carbon content was 19–31 fg C cell^−1^. Based on counted cell numbers (Andrén et al., 2015; Supplementary Table 1), the mean carbon content of total microbial cells in the sediment decreased from 350 μg C cm^−3^ sediment to < 5 μg C cm^−3^ sediment (Figure 4).

**Table 2:** **Cellular content of THAA, THAA-C, and total C in cell extract samples from Landsort Deep, Baltic Sea**.

**Sediment depth (mbsf)**	**THAA (fmol cell^−1^)**	**THAA-C (fmol cell^−1^)**	Total C^*^ (fg C cell^−1^)
0.4	0.18	0.88	19
2.75	0.29	1.2	26
4.32	0.28	1.3	29
9.57	0.31	1.4	31
14.55	0.21	0.97	21
20.53	0.13	0.66	14
38.95	0.17	0.78	17

* *Calculated from total amino acid-carbon (THAA-C) assuming that THAA-C = 55% of total cell-carbon (Ingraham et al., 1983)*.

**Table 3:** Mole percentage composition for each hydrolysable amino acid in cell extract samples from Landsort Deep, Baltic Sea^*^.

**Depth (mbsf)**	**Mole %**																	
	**Asp**	**Glu**	**Ser**	**His**	**Gly**	**Thr**	**Arg**	**β-Ala**	**Tau**	**Ala**	**γ-Aba**	**Tyr**	**Val**	**Phe**	**Ileu**	**Leu**	**Orn**	**Lys**
0.4	5.8	8.5	8.1	0.9	11.7	9.1	5.1	n.d.	n.d.	14.2	n.d.	3.1	8.4	5.0	4.4	10.3	n.d.	5.2
2.75	5.0	8.4	13.0	0.7	31.5	5.2	3.7	n.d.	n.d.	8.4	n.d.	2.8	4.4	3.4	2.2	6.7	n.d.	4.7
4.32	4.5	8.8	10.5	0.9	14.0	6.8	5.8	n.d.	n.d.	10.6	n.d.	3.6	6.9	3.6	10.0	8.8	n.d.	5.2
9.57	9.4	12.3	11.8	1.0	18.3	4.7	4.7	n.d.	n.d.	7.6	n.d.	3.2	5.0	3.5	3.0	10.2	n.d.	5.4
14.55	5.8	7.5	7.7	0.6	13.2	8.5	4.4	n.d.	n.d.	16.0	n.d.	2.8	8.5	5.0	4.0	10.3	n.d.	5.9
20.53	10.9	10.7	5.1	0.9	7.5	7.2	4.6	n.d.	n.d.	16.0	n.d.	2.9	8.8	4.9	3.8	12.0	n.d.	4.7
38.95	10.0	14.1	11.7	1.2	14.4	4.7	5.2	n.d.	n.d.	6.7	n.d.	3.4	5.5	3.8	1.3	12.5	n.d.	5.5

* *Asp, aspartic acid; Glu, glutamic acid; Ser, serine; His, histidine; Gly, glycine; Thr, threonine; Arg, arginine; β-Ala, β-alanine; Tau, taurine; Ala, alanine; γ-Aba, γ-aminobutyric acid; Tyr, tyrosine; Val, valine; Phe, phenylalanine; Ileu, isoleucine; Leu, leucine; Orn, ornithine; Lys, lysine; n.d., not detected*.

**Figure 5:**
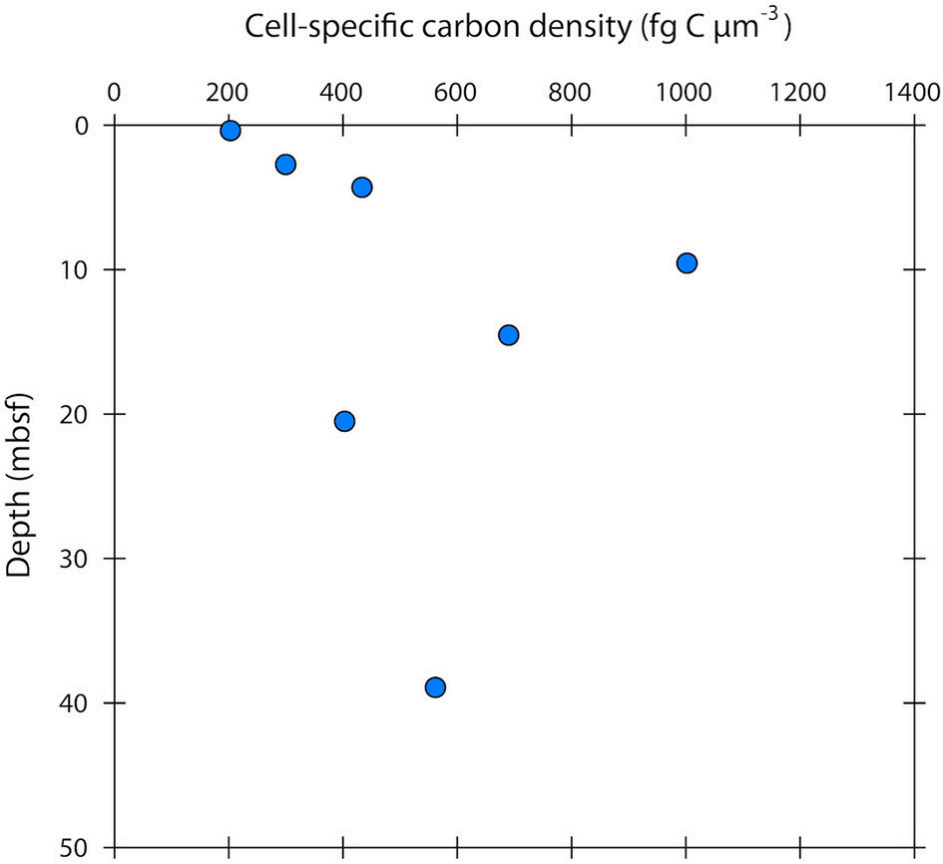
**Cell-specific carbon density in sub-seafloor microbial cells at Landsort Deep, Baltic Sea**. The mean cell-specific carbon density (in the literature often expressed as “carbon-volume ratio”) increases with depth, indicating that cells become more “packed” and “dry” by decreasing their water content.

The cell-specific carbon density increased with sediment depth from ~200 to 600 fg C μm^−3^, peaking at 1000 fg C μm^−3^ at 10 m depth (Figure 5).

## Discussion

### Cell-specific carbon content and carbon density

The organic carbon content of bacteria and archaea (mainly from proteins, RNA, lipids, and polysaccharides) can differ strongly among environments and cultures (e.g., Bratbak, 1985; Kogure and Koike, 1987; Simon and Azam, 1989; Fukuda et al., 1998; Lever et al., 2015). For the deep biosphere, global abundance and biomass of microbial cells have been estimated from the available data on cell abundances in the marine subsurface. The first global extrapolation indicated that 94% of all microbial cells inhabit the deep biosphere and account for a third to half of all living biomass carbon on Earth (Whitman et al., 1998). Based on a larger data set that also included very low cell abundances for the large oligotrophic ocean gyres, Kallmeyer et al. (2012) downscaled these numbers to a global inventory of 2.9 × 10^29^ cells, corresponding to 0.18–3.6% of total global biomass. However, these biomass estimates were not based on direct measurements of the carbon content of sub-seafloor microbial cells. Instead, they were based on mean cellular carbon contents that were deduced from, for example, cell dry weight (Whitman et al., 1998), sedimentary lipid biomarker concentrations (Lipp et al., 2008), or cell volumes (Kallmeyer et al., 2012). While the cell volumes and the lipid concentrations were quantified from actual sub-seafloor samples, the data for the dry weight consisted of one single data point obtained from a terrestrial aquifer (Balkwill et al., 1988). These parameters were then converted into cellular carbon contents using relationships determined for pure cultures or planktonic bacteria from water samples (e.g., Norland et al., 1987; Balkwill et al., 1988; Simon and Azam, 1989).

**Figure 6:**
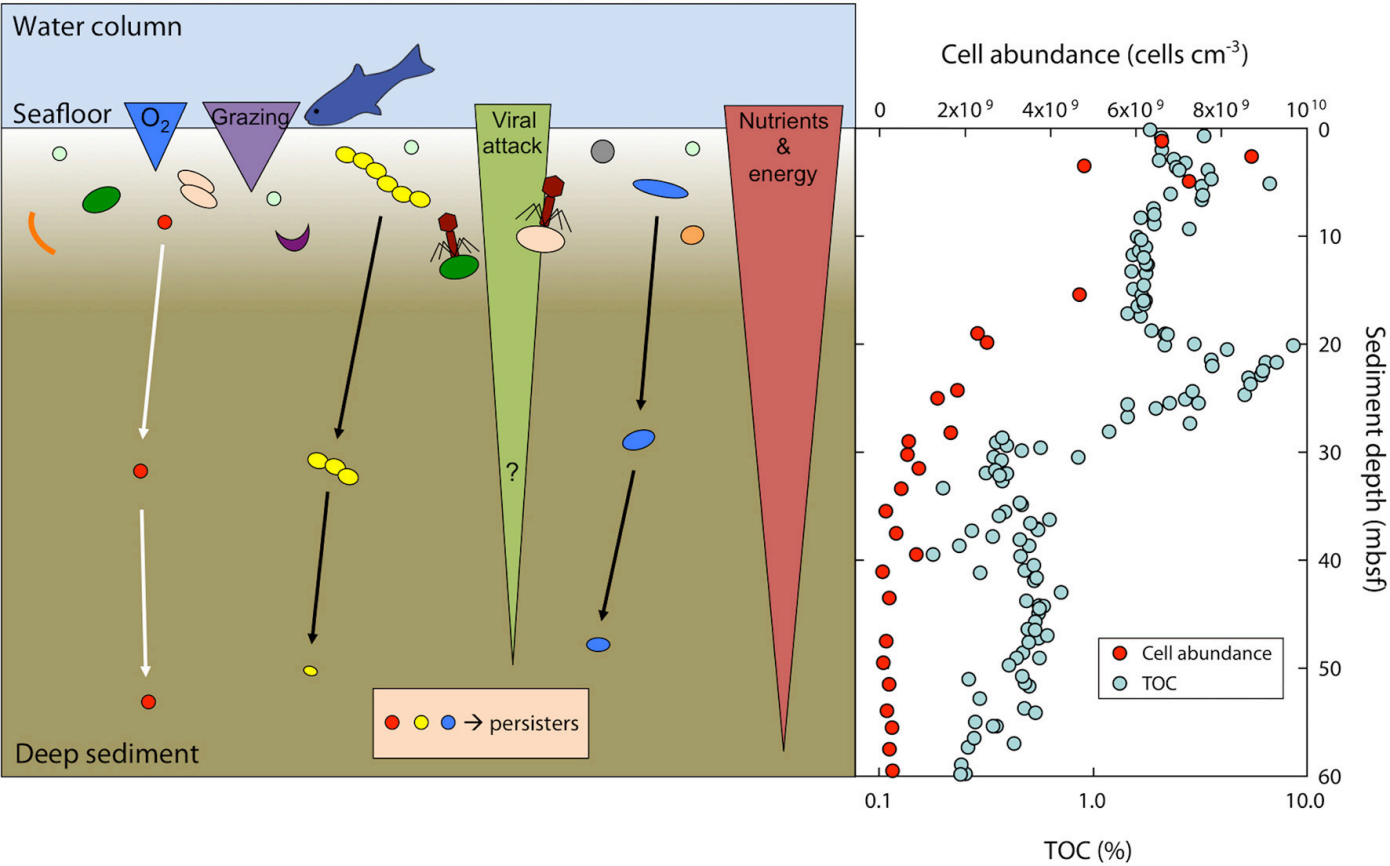
**Scheme of the survival and size-adaptation of cells during burial in marine sediments**. Typically, about 90% of the surface community dies within a few mm to cm of burial due to a variety of factors such as predation (grazing), viral attack, oxygen levels, toxic waste products, or nutrient and energy limitation (left panel). A small subset of the surface population is able to survive at the harsh conditions faced during burial and will further adapt by growing small cell sizes to reduce maintenance costs. Note that bacterial size and their absolute abundance are not to scale. Total cell abundance (acridine orange direct counts, AODC) is steeply decreasing in sediments at Landsort Deep (right panel). Total organic carbon (TOC) deposited from the surface photosynthetic world serves as electron donor and energy source and is decreasing with sediment depth at Landsort Deep (right panel). Data for cell abundance and TOC taken from Andrén et al. (2015).

Our cell extraction and purification procedure allowed us to directly determine the amino acid composition and amino acid-carbon content of the cell extracts. Purification of cell extracts by FACS was important to remove cells from remaining detrital organic particles after Nycodenz-based density centrifugation (Braun et al., 2016). Total cell carbon was then estimated for all samples by assuming that amino acids contain 55% of total cell carbon (Ingraham et al., 1983). The cell-specific carbon content in our samples from Landsort Deep ranged from 19 to 31 fg C cell^−1^, with a mean value of 23 fg C cell^−1^. This estimate is at the lower end of those for eutrophic estuarine and coastal systems (Bratbak, 1985; Bjørnsen, 1986; Kogure and Koike, 1987; Lee and Fuhrman, 1987; Kroer, 1994), but similar to those for starved laboratory cultures or the oligotrophic open ocean (e.g., Simon and Azam, 1989; Fukuda et al., 1998). Notably, our new estimate is 3–4 times lower than widely used previous estimates for marine sediments of 65 fg C cell^−1^ (Parkes et al., 1994) and 86 fg C cell^−1^ (Whitman et al., 1998) and 6 times lower than the carbon content of cells sorted from an *E. coli* culture (Braun et al., 2016; Figure 4), which was analyzed in the same way as the samples from this study. However, our new estimate is higher than the 14 fg C cell^−1^ used for the most recent global estimate of microbial biomass (Kallmeyer et al., 2012). The estimate of Kallmeyer et al. (2012) was based on cell volumes determined by fluorescence microscopy for sediment samples from the oligotrophic South Pacific Gyre. The conversion from volumes to carbon contents was established from relationships between cell size and protein content of pelagic bacteria (Simon and Azam, 1989), which may not be appropriate for the deep biosphere. A mean value of 14 fg C cell^−1^, however, may indicate that cells that live under energy-deprived conditions have a lower cell-specific carbon content than cells that live in environments with higher amounts of available energy from organic matter. At Landsort Deep, TOC concentrations decrease from ~3% at the sediment surface to ~0.2% at 60 mbsf (Figure 6). In contrast, TOC concentrations in the South Pacific Gyre are ~0.3–0.5% at the sediment surface and slowly decline to < 0.1% at 5 mbsf. Our estimate and that of Kallmeyer et al. (2012) seem very close to each other when considering the enormous difference of the sediment ages. Due to the low sedimentation rates of < 0.1 cm kyr^−1^ in the South Pacific Gyre (D'Hondt et al., 2009), the sediment age at a depth of 5–10 mbsf is 25–100 million years (D'Hondt et al., 2009). At Landsort Deep, sedimentation rates are 100–500 cm kyr^−1^, and the age of the sediment column investigated here is < 15,000 years (Andrén et al., 2015). Thus, it appears that the cell-specific carbon content in marine sediments is a relatively constant parameter, possibly because the cells require a minimum set of macromolecules to remain functional.

Our assumption that amino acids contain 55% of total cell carbon (Ingraham et al., 1983) does not change the relative differences between the cell-specific (amino acid-) carbon contents between the samples since we used the same assumption for all of the samples. However, multiplying cellular amino acid-carbon (AA-C) by a factor of 1/0.55 = 1.82 is based on cultures of *E. coli* (Ingraham et al., 1983). Cells in the deep biosphere may have a different AA-C/total-C ratio than cultured *E. coli* cells. Even though literature about these ratios in prokaryotes from environmental sample is scarce, we suggest that the AA-C/total-C ratio of 55% is appropriate based on the study by Simon and Azam (1989). The authors studied cell sizes and protein (amino acids) contents in pelagic bacteria and—based on the cellular fractions of other macromolecular pools such as the cell wall, cell membrane, and nucleic acids—estimated cell dry weight and total cell carbon. Throughout the samples, the cell protein:dry weight and cell carbon:dry weight ratios were essentially constant (63 and 54%, respectively), although mean cell diameters ranged over one order of magnitude from 0.026 to 0.4 μm. Most importantly, cell carbon had a constant relationship with cell protein. On average, the ratio between cellular amino acid-carbon and total cell-carbon was 61%, which is only slightly higher than the 55% we have used for our samples.

If the amino acids in the cells investigated in this study contained more than 55% of total cell-C, the total cell-specific carbon contents would be lower than the values reported here. For example, when we assume that AA-C makes up 75% (instead of 55%) of total cell-C in the deepest of our samples, the cell-specific carbon content would be 13 fg C cell^−1^ (instead of 17 fg C cell^−1^). Yet, the difference of 4 fg C cell^−1^ is still small compared to the differences to the widely used previous estimates for marine sediments of 65 fg C cell^−1^ (Parkes et al., 1994) and 86 fg C cell^−1^ (Whitman et al., 1998). Since protein is a major macromolecule with essential functions in cells, it is unlikely that it makes up only a small fraction of the cell biomass. However, a AA-C/total cell-C ratio of, for example, 40% would result in a cell-specific carbon content of 23 fg C cell^−1^ for the deepest investigated sample in this study. Even though this value exceeds our estimate by 6 fg C cell^−1^, it is still much lower than the estimates by Parkes et al. (1994) and Whitman et al. (1998). Notably, the mean cell-specific carbon content of 14 fg C cell^−1^ that was used for the most recent global microbial biomass estimate by Kallmeyer et al. (2012) seems to be a realistic estimate of the carbon content of deep biosphere cells.

Our new estimates of the mean cell-specific carbon content will also be important for estimating microbial generation times in the deep biosphere (Langerhuus et al., 2012; cf. Lomstein et al., 2012; Hoehler and Jørgensen, 2013). These are typically calculated from cell-specific carbon assimilation rates and assumed cellular carbon contents and growth yields. Carbon assimilation rates are often calculated from activity measurements using radiotracers such as ^35^S-labeled sulfate for sulfate reduction and ^14^C-labeled bicarbonate or acetate for methanogenesis (e.g., Parkes et al., 1990). Cells with a high cell-specific carbon content need to assimilate more carbon to renew all cell-carbon than those cells that have low carbon contents. Cell-carbon turnover times are in the order of years to hundreds of years when an elevated cell-specific carbon content of 88 fg C cell^−1^ (Langerhuus et al., 2012; Lomstein et al., 2012) or 65 fg C cell^−1^ (Hoehler and Jørgensen, 2013) is used. Using our new mean estimate of 23 fg C cell^−1^, turnover times will be 3-4 times lower than the previous estimates.

Few studies have measured cell-specific carbon contents in low energy environments, and these have shown low values with a small range of variation as we have shown here (Fukuda et al., 1998; Vrede et al., 2002). Biovolume decrease and cell shrinkage are more commonly used indications of energy limitation (Lever et al., 2015). Concurrently, the cell-specific carbon density has been shown to increase upon starvation in various cultured marine and non-marine strains (Nagata and Watanabe, 1990; Troussellier et al., 1997). The cell-specific carbon density that we observed in our cell extracts increased with sediment depth from ~200 to 600 fg C μm^−3^, peaking at 1000 fg C μm^−3^ at 10 m depth (Figure 5). These carbon densities are consistent with those reported from natural bacterial assemblages of 130–1600 fg C μm^−3^ (Bratbak, 1985; Bjørnsen, 1986; Lee and Fuhrman, 1987; Simon and Azam, 1989; Kroer, 1994). For comparison, the carbon density in growing *E. coli* cells, and in several other growing cultures, is typically < 100 fg C μm^−3^ (Fagerbakke et al., 1996). The cell-specific carbon density has been shown to increase with decreasing cell volume (Simon and Azam, 1989; Troussellier et al., 1997), but also seems to be species dependent and greatly affected by energy conditions (Troussellier et al., 1997). Elevated cell-specific carbon densities imply low cellular water contents. Simon and Azam (1989) reported that marine diminutive bacteria with volumes around 0.03 μm^3^ had a very low water content of only 46% (v:v), whereas larger bacteria (0.4 μm^3^) had a high water content of 82% (v:v). Interestingly, the cell carbon:dry weight ratio was essentially constant throughout the range of cell sizes. Microbes with diminutive volumes might therefore change their cell size by disproportionally varying the water content as compared to the biomass, thus reducing energetic costs (Simon and Azam, 1989). Given the large range of variation in the cell-specific carbon density, cell-specific carbon contents obtained solely from measured cell volumes, and the use of general conversion factors can have large error bars.

### Cell volume

Microorganisms commonly found in natural environments such as soils, freshwater lakes and rivers, estuarine and brackish waters, coastal and nearshore marine waters, or pelagic marine waters generally have cell volumes between 0.05 and 0.5 μm^3^ (cf. reviews by Romanova and Sazhin, 2010; Lever et al., 2015). These volumes are typically 2–10-fold lower than those of cells in growing pure cultures (e.g., Trueba and Woldringh, 1980; Montesinos et al., 1983; Troussellier et al., 1997). The mean cell volumes in sediments at Landsort Deep are 0.007–0.095 μm^3^, which is up to an order of magnitude lower than those reported for microbes in freshwater lakes or energy-rich coastal waters and are closer to those from the oligotrophic open ocean (Børsheim, 1990). The volumes are similar to the mean cell volume of 0.042 μm^3^ in deep old marine sediments determined by Kallmeyer et al. (2012). The energy-deprived deep biosphere is characterized by selective pressure (Starnawski, 2016) and extremely low metabolic rates (e.g., D'Hondt et al., 2004; Schippers et al., 2005; Jørgensen and D'Hondt, 2006; Langerhuus et al., 2012; Lomstein et al., 2012; Røy et al., 2012) that allow cell division only on annual to centennial timescales (reviewed in Hoehler and Jørgensen, 2013; Jørgensen and Marshall, 2016). Cells from pure cultures typically respond to experimental energy limitation by reducing their cell size and volume within days to weeks, either by shrinking (Novitsky and Morita, 1976; Kieft et al., 1997) or by fragmentation, that is, cell division without growth (Amy and Morita, 1983; Amy et al., 1993). When considering the physiological state of cells in the energy-limited, but extremely stable deep biosphere, comparisons with acute stress responses of cultured cells are, however, misleading. Cells in the deep biosphere live under balanced conditions at extremely low metabolic rates, comparable to a chemostat culture that grows for many generations under constant conditions over weeks or months (Hoehler and Jørgensen, 2013; Jørgensen and Marshall, 2016). The cells are in physiological balance (i.e., stable growth rate, unchanging macromolecular composition such as the composition of membrane lipids, etc.) while being energetically constrained in their metabolic rate. About 90% of the surface community dies within a few mm to cm of burial (e.g., Langerhuus et al., 2012; Figure 6) due to a variety of factors such as predation, viral attack, oxygen levels, toxic waste products, or nutrient and energy limitation. A small subset of the surface population is, however, pre-adapted to the harsh conditions faced during burial (Starnawski, 2016). These cells survive burial and will further adapt to the environmental conditions during burial (Figure 6). In deep sediments, the community size decreases only marginally (<1%) per generation (Jørgensen and Marshall, 2016), and cells are slowly selected for properties that enhance nutrient uptake, facilitate ATP synthesis, reduce energy loss (e.g., leakage of protons through the cell membrane), and counteract biomolecule decay over thousands to millions of years. Since cells in the deep biosphere are predominantly very small (i.e., < 0.05 μm^3^), they may have been selected for because small “packed” cells with a low water content (see also Section Cell-Specific Carbon Content and Carbon Density) may be less prone to intracellular damage from chemical reactions such as amino acid racemization, protein denaturation, or DNA depurination. The cells may also actively adapt to the energy-limiting conditions by growing small cell sizes to minimize maintenance requirements (Oliver, 1993; Figure 6). Furthermore, subcellular architecture might be preserved due to the protective, glass-like properties of the bacterial cytoplasm at low metabolic activity (Parry et al., 2014).

The reduction in cell size leads to an increased surface-to-volume ratio. In the oligotrophic water column, cells often increase their surface-to-volume ratio to facilitate the uptake of scarce substrates (Gottschal, 1992). In the deep biosphere, however, it is unlikely that the reduction in cell size considerably affects the uptake of substrates because they are turned over very slowly. The transport over the cell wall, and thereby the surface-to-volume ratio itself, is probably not the main competitive control. Glombitza et al. (2015) calculated that the turnover and diffusion times of volatile fatty acids (VFAs, e.g., acetate) at several meters depth in sediments offshore southwest Greenland. While VFA diffusion times were short (below 0.5 s), their turnover times were up to a year, showing that diffusion is not limiting VFA turnover. Under these conditions, cell physiological constraints such as the energetic costs of VFA activation or uptake may be a limiting factor in substrate turnover rather than kinetic constraints due to cell surface area or cell volume.

Even though observed differences in cell volumes may arise due to a variety of environmental factors such as energy, nutrients, and redox conditions, they may also be the result of methodological problems. While a large body of data exists from around the World Ocean, based on fluorescence microscopy of DNA stained cells, a number of studies have addressed advantages and disadvantages of cell size estimations with FM or SEM (cf. Fuhrman, 1981; Bratbak, 1993; Bölter et al., 2002; Malfatti et al., 2010; Romanova and Sazhin, 2010). These studies usually included recommendations for sample treatments and analyses to account for measurement artifacts. For example, fluorescent latex spheres of known diameter have widely been used to calibrate sizes of objects that are surrounded by aureoles (e.g., Bratbak, 1993; Loferer-Krößbacher et al., 1998; Pelegri et al., 1999). Size measurements of sub-micrometer microbial cells within an environmental matrix ideally involve a fluorescent label for clear identification of cells, combined with the spatial resolution of a scanning electron microscope. Even though it is possible to combine SEM or AFM with FM for better identification of cells, several correction factors need to be used in order to account for the remaining artifacts from sample preparations. In our study, we used correction factors obtained from the literature or from experiments with cultured cells. Since cultured cells may show different responses to sample treatments than deep biosphere cells, the appropriateness of the correction factors can be questioned. For example, the mean literature value used to correct for dehydration and CPD was based on cultured cells (Bratbak, 1985), which probably had a higher water content than deep biosphere cells and may have been more prone to shrinkage upon dehydration. However, since the corrected FM- and SEM-based cell volumes in our study do compare, we conclude that the correction factors were appropriate. Super-resolution fluorescence microscopy that operates beyond the diffraction barrier imposed by the wave nature of light, such as photoactivated localization microscopy (PALM), stochastic optical reconstruction microscopy (STORM), and stimulated emission depletion (STED) microscopy (cf. Hell, 2009; Coltharp and Xiao, 2012), have not been used in deep biosphere research yet. However, these techniques could—upon the development of suitable fluorophores for cell tagging (Klar et al., 2000)—overcome problems faced with FM, SEM, or AFM, and may reduce the need for correction factors.

## Conclusion

Our data showed that average cell volumes decreased with sediment depth by up to one order of magnitude and were 10–100 times smaller than those of growing *E. coli* cells. Based on measurements of cellular amino acids, estimates of the cell-specific carbon content were 19–31 fg C cell^−1^. The data verifies a low but relatively constant cell-specific carbon content as a general feature of subsurface microbial life. The cell-specific carbon density was increasing with sediment depth and therefore the water content was decreasing. Microbial communities in the deep biosphere grow and multiply with extremely low rates of metabolism. Growing small cell sizes (i.e., in this context, < 0.05 μm^3^) seems to be one of probably many adaptations of sub-seafloor microbial life to energy limitation.

## Author contributions

SB, MD, SL, MK, BJ, and BL designed the study; SB, YM, SL, and HA performed the laboratory work; SB and HA performed data analysis; SB wrote the manuscript; BL, BJ, YM, SL, and MK edited the manuscript.

### Conflict of interest statement

The authors declare that the research was conducted in the absence of any commercial or financial relationships that could be construed as a potential conflict of interest.
