# Supplementary material for: Size and Carbon Content of Sub-seafloor Microbial Cells at Landsort Deep, Baltic Sea
Source: Front Microbiol. 2016 Aug 31;7:1375. doi: 10.3389/fmicb.2016.01375 (PMC5005352; doi:10.3389/fmicb.2016.01375)
Supplement: Supplementary file 1 [file DataSheet1.DOCX]

Supplementary Material

Biovolume and carbon content of sub-seafloor microbial cells at Landsort Deep, Baltic Sea

Stefan Braun^1*^, Yuki Morono^3^, Sten Littmann^4^, Hüsnü Aslan^5^, Mingdong Dong^5^, Bo B. Jørgensen^1^, Bente Aa. Lomstein^1,2^

^1^Center for Geomicrobiology, Department of Bioscience, Aarhus University, Aarhus, Denmark

^2^Section for Microbiology, Department of Bioscience, Aarhus University, Aarhus, Denmark

^3^Geomicrobiology Group, Kochi Institute for Core Sample Research, Japan Agency for Marine-Earth Science and Technology (JAMSTEC), Kochi, Japan

^4^Biogeochemistry Group, Max Planck Institute for Marine Microbiology, Bremen, Germany

^5^Interdisciplinary Nanoscience Center (iNANO), Aarhus University, Aarhus, Denmark

*** Correspondence:** Stefan Braun: stefan.braun@bios.au.dk

# Calculations of cell volumes

For the calculation of the FM- and SEM-based cell volumes, we used the following formulas:

(1) $V_{coccus}= \frac{4}{3}\pi r^{3}$

for coccoid cells (spheres).

(2) $V_{rod}= \pi r^{2}\times h+\frac{4}{3}\pi r^{3}$

for rod-shaped cells (hemisphere-capped cylinders), where h = length – 2*r*.

(3) $V_{prolate spheroid}= \frac{4}{3}\pi a^{2}b$

for prolate spheroids, where *a* is the length of the minor axis of the cell, and *b* is the length of the major axis. (Note that prolate spheroids could only be identified by SEM, but not by FM due to insufficient optical resolution. With FM, elongated cells were treated as rod-shaped cells.)

(4) $V_{filament}=\pi r^{2}\times h$

for filamentous cells (cylinders; length:width ratio >10), where *h* is the length of the cell.

For the calculation of the AFM-based cell volumes, we used the formula

(5) $V_{ellipsoid}= \frac{4}{3}\pi\times a\times b\times c$

which takes into account the height of the cells (cf. Malfatti et al., 2010), where *a*, *b*, *c* are semi-axes of the solid ellipse (*a* = length/2; *b* = width/2; *c* = height/2).

# Supplementary Figures and Tables


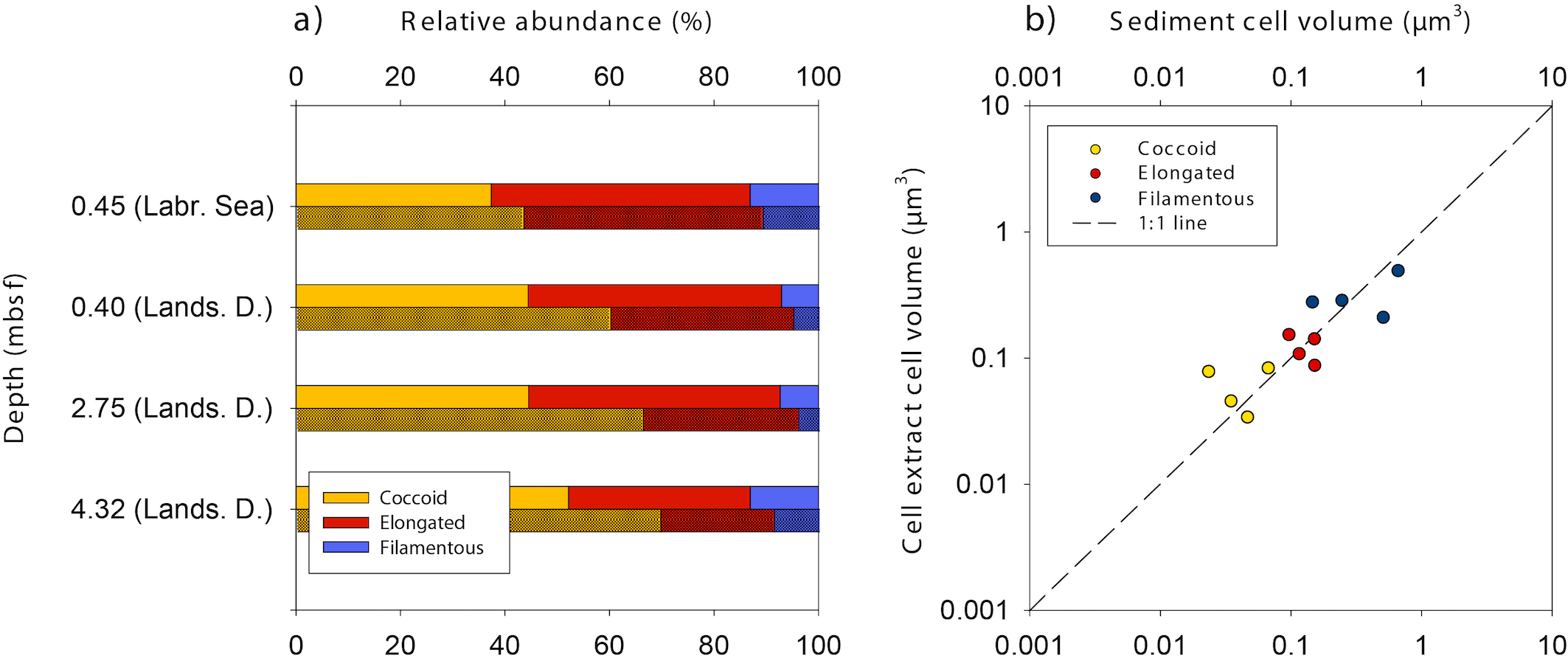


**Supplementary Fig. 1** **Comparison of cell shapes and volumes of extracted cells to those in whole sediment in three samples from Landsort Deep and one sample from the Labrador Sea.** **a**, Cell morphology is similar between extracted and non-extracted cells, but the relative amount of coccoid cells is slightly higher in the sediments than in the cell extracts. Small coccoid cells might be able to more firmly attach to mineral grains than elongated cells, and therefore they could be lost into the sediment pellet during density centrifugation. Data obtained with FM (lighter shade) and SEM (darker shade). **b**, The cell volumes of extracted and non-extracted cells followed a 1:1 line, indicating that extraction of cells from sediment was representative in terms of size.


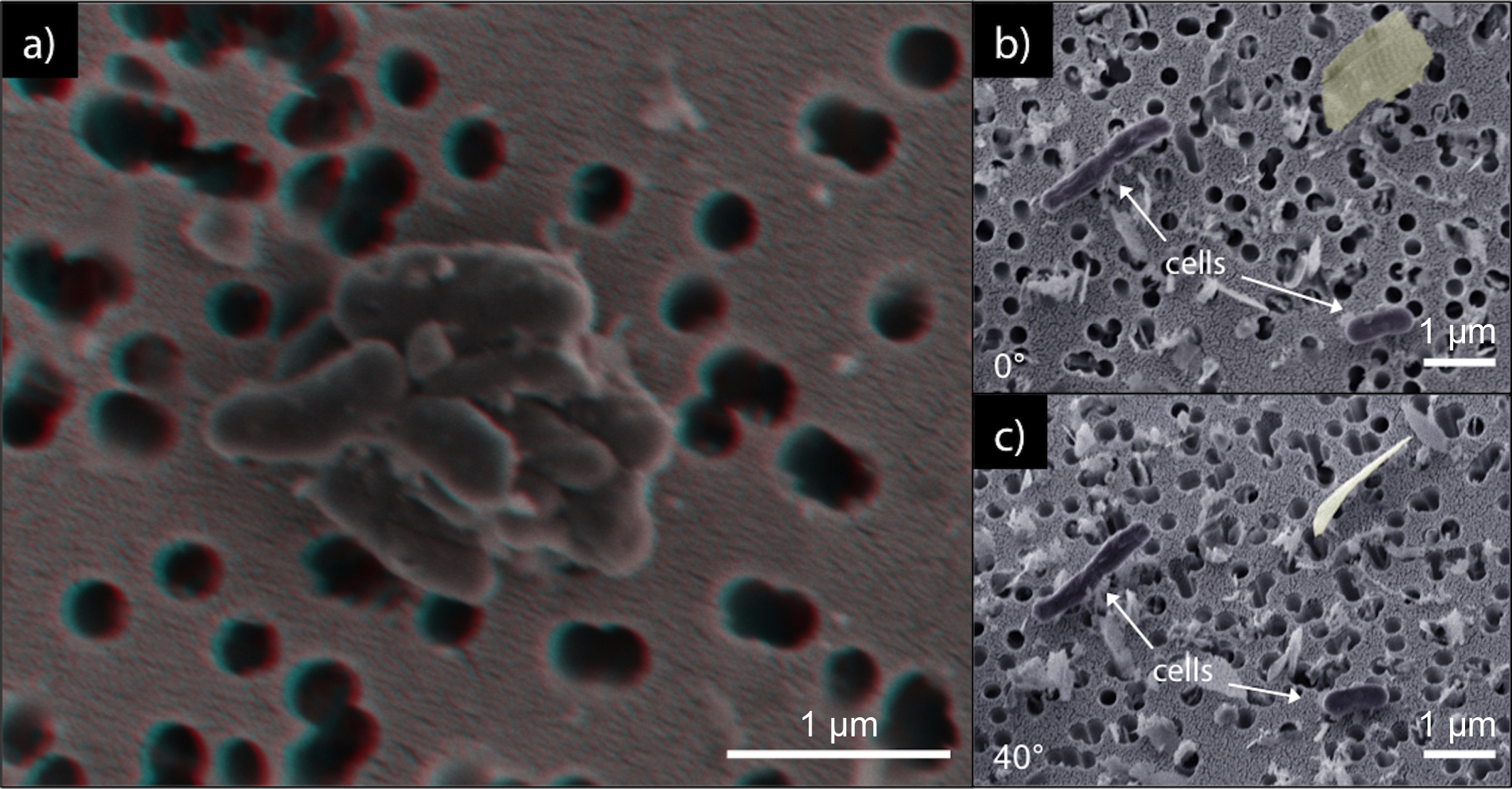


**Supplementary Fig. 2** **Effect of critical point drying on cell integrity**. After dehydration with ethanol followed by critical point drying, cells retained their full 3-dimensional shape, as exemplified here on a cell aggregate (**a**, imaged as a stereoscopic image best viewed with 3-D red-cyan glasses) and on two rod-shaped cells that do not show any flattening (**b,c**). Note that for imaging the two rod-shaped cells (violet), the sample table was tilted from 0º (**b**) to 40º (**c**), which is clearly visible from the rotation of a flat detrital particle (yellow).


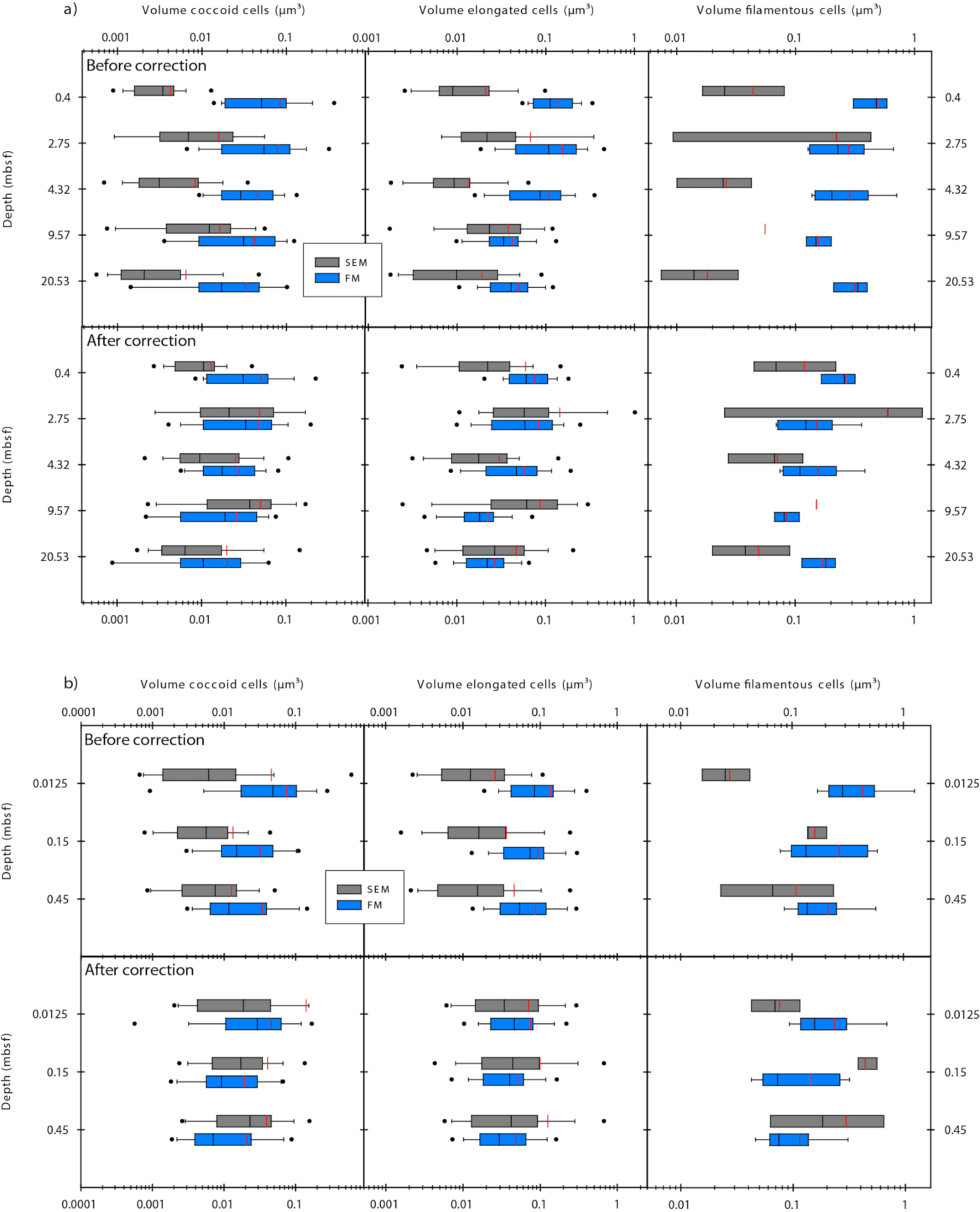


**Supplementary Fig. 3** **Cell volumes determined with epifluorescence microscopy (FM) and scanning electron microscopy (SEM) before and after correction for cell fixation, filtration, dehydration and critical point drying, and the fluorescence-halo effect.** After correction, cell volumes obtained with FM were similar to those obtained with SEM. **a**, Samples from Landsort Deep; **b**, samples from the Labrador Sea off SW Greenland. Red line indicates mean value, black line indicates median value. The blue boxes indicate the 25^th^/75^th^ percentile. Whiskers show 10^th^/90^th^ percentile, dots show 5^th^/95^th^ percentile.

**Supplementary Table 1:** Cell counts obtained from linear interpolation of acridine orange direct counts (AODC, Andrén et al., 2015) at adjacent depths.

| **Sediment depth (mbsf)** | **Cells cm^-3^** |
| --- | --- |
| 0.4 | 1.80 × 10^10^ |
| 2.75 | 8.26 × 10^9^ |
| 4.32 | 6.16 × 10^9^ |
| 9.57 | 6.11 × 10^9^ |
| 14.55 | 4.90 × 10^9^ |
| 20.53 | 2.41 × 10^9^ |

**References**

Andrén, T., Jørgensen, B.B., Cotterill, C.; Green, S., and Exped. 347 Sci. (2015) *Proceedings of the Integrated Ocean Drilling Program*, Vol. 347: *Baltic Sea Paleoenvironment*. Tokyo: Integr. Ocean Drill. Program Manag. Int., **http://publications.iodp.org/proceedings/347/347title.htm**
